# Synthesis of Novel 2,5-Disubstituted-1,3,4-thiadiazoles Clubbed 1,2,4-Triazole, 1,3,4-Thiadiazole, 1,3,4-Oxadiazole and/or Schiff Base as Potential Antimicrobial and Antiproliferative Agents

**DOI:** 10.3390/molecules200916048

**Published:** 2015-09-02

**Authors:** Nadjet Rezki, Amjad M. Al-Yahyawi, Sanaa K. Bardaweel, Fawzia F. Al-Blewi, Mohamed R. Aouad

**Affiliations:** 1 Department of Chemistry, Faculty of Sciences, Taibah University, P. O. Box 344, Al-Madinah Al-Munawarah 30002, Saudi Arabia; E-Mails: mjooooda_88@hotmail.com (A.M.A.-Y.); ffs_chem334@hotmail.com (F.F.A.-B.); 2 Laboratoire de Chimie & Electrochimie des Complexes Métalliques (LCECM) USTO-MB, Department of Chemistry, Faculty of Sciences, University of Sciences and Technology Mohamed Boudiaf, P. O. Box 1505, El M`nouar, Oran 31000, Algeria; 3 Department of Pharmaceutical Sciences, Faculty of Pharmacy, University of Jordan, Amman 11942, Jordan; E-Mail: S.Bardaweel@ju.edu.jo

**Keywords:** 1,3,4-thiadiazole, 1,2,4-triazole, 1,3,4-oxadiazole, Schiff base, antimicrobial activity, antitumor activity

## Abstract

In the present study, a new series of 2,5-disubstituted-1,3,4-thiadiazole tethered 1,2,4-triazole, 1,3,4-thiadiazole, 1,3,4-oxadiazole and Schiff base derivatives were synthesized and characterized by IR, ^1^H-NMR, ^13^C-NMR, MS and elemental analyses. All compounds were screened for their antibacterial, antifungal and antiproliferative activity. Some of the synthesized derivatives have displayed promising biological activity.

## 1. Introduction

Aromatic five-membered nitrogen heterocycles have been potential targets of investigations by several research groups owing to their interesting biological activities and medicinal properties [1,2]. Among these, the 1,2,4-triazole scaffold constitutes the core moiety of several therapeutically active compounds as antimicrobial [3], analgesic [4], antiviral [5], antioxidant [6], anti-inflammatory [7], and anticancer [8] agents. Furthermore, 1,3,4-thiadiazoles and 1,3,4-oxadiazoles are also important classes of azoles endowed with significant biological properties as there are several examples in the literature including antifungal [9,10], anti-inflammatory [11,12], antimicrobial [13,14], antiviral [15,16] and anticancer [17,18] activities. Additionally, many investigations showed that the clubbing of two or three heterocyclic units may significantly potentiate the antimicrobial activities [19,20,21]. In addition, Schiff bases have been the focus of numerous studies due to their wide spectrum of biological activities [22]. Moreover, azomethine Schiff bases linkages, as attractive connecting units that could bind two pharmacophores to generate an innovative bifunctional drugs, have rapidly emerge as one of the most challenging and attractive topics in drug design for the constructing of novel bioactive molecules. Based on all above considerations and as an extension of our studies on the developments of novel azoles antimicrobial agents [23,24], we disclosed the synthesis of new polyheterocyclic ring systems with anticipated antimicrobial and antiproliferative activities, by clubbing 1,3,4-thiadiazole with 1,2,4-triazole, 1,3,4-thiadiazole, 1,3,4-oxadiazole and/or Schiff base moiety in one frame work.

## 2. Results and Discussion

### 2.1. Chemistry

**Scheme 1:**
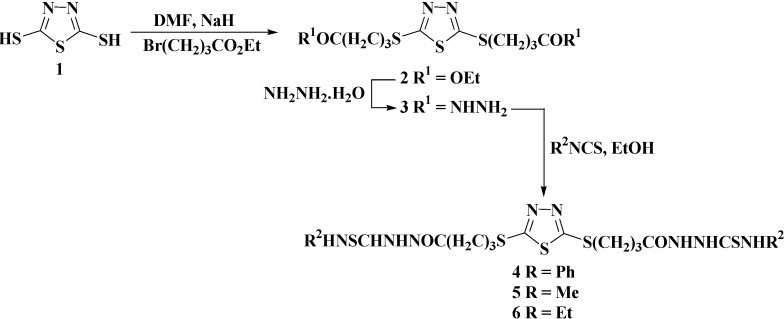
Synthesis of bis-acid thiosemicarbazides **4**–**6**.

**Scheme 2:**
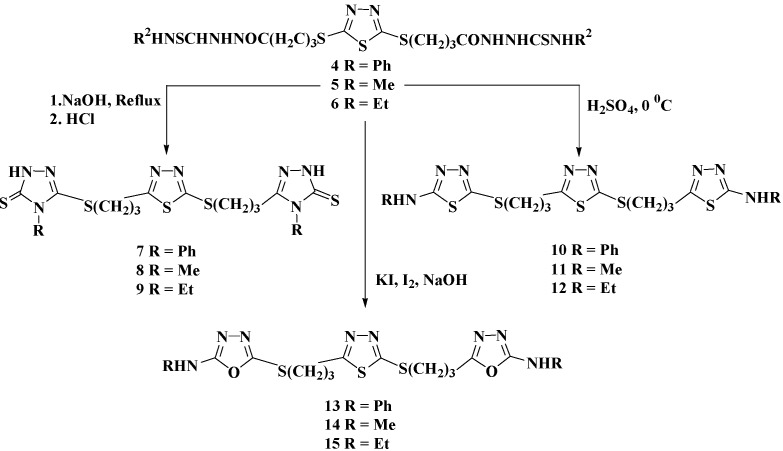
Synthesis of 1,2,4-triazoles **7**–**9**, 1,3,4-thiadiazoles **10**–**12** and 1,3,4-oxadiazoles **13**–**15**.

**Scheme 3:**
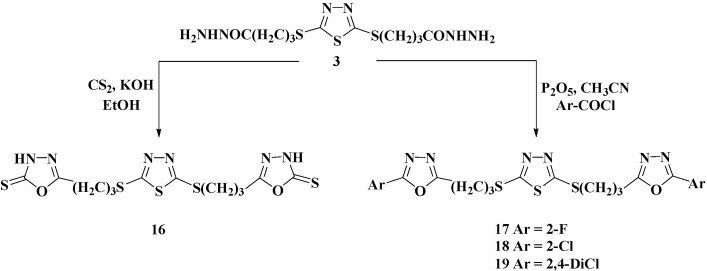
Synthesis of 1,3,4-oxadiazole derivatives **16**–**19**.

**Scheme 4:**
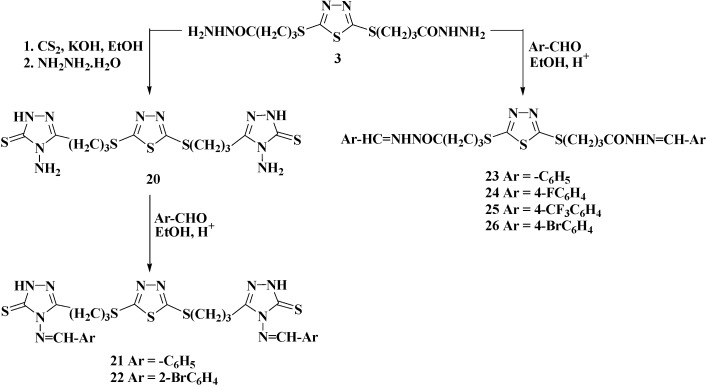
Synthesis of 4-amino-1,2,4-triazole **20** and Schiff bases **21**–**26**.

The target azoles were prepared started from 2,5-dimercapto-1,3,4-thiadiazole (**1**) [25] via multi-step synthesis as illustrated and outlined in Scheme 1, Scheme 2, Scheme 3 and Scheme 4.

The alkylation of 2,5-dimercapto-1,3,4-thiadiazole (**1**) with 4-ethylbromobutyrate in dimethylformamide in the presence of potassium carbonate as base gave diethyl 4,4′-[(1,3,4-thiadiazol-2,5-diyl)bis(sulfanediyl)]dibutanoate (**2**) in 92% yield (Scheme 1). The structures of the newly synthesized compounds were characterized by IR, ^1^H-NMR, ^13^C-NMR, Mass spectra and elemental analysis. Compound **2** exhibited NMR and IR spectra congruent with its assigned structure. Its IR spectrum showed strong absorption bands at 1740 and 1270 cm^−1^ due to (C=O) and (C-O) groups of ester **2**, respectively. In addition, the appearance of two absorption bands at 2879 and 2936 cm^−1^ corresponding to the aliphatic (C-H) streching vibrations confirm the success of the alkylation reaction. In the ^1^H-NMR spectrum, the characteristic ester protons (C**H_2_**C**H_3_**) resonated as triplet at δ_H_ 2.06 ppm and quartet at δ_H_ 4.08–4.12 ppm. The butyric methylene protons CH_2_C**H_2_**CH_2_, C**H_2_**CO and SC**H_2_** were observed as two multiplets at δ_H_ 1.78–1.82 and 1.87–1.93 ppm and triplet at δ_H_ 3.32 ppm, respectively. Moreover, the ^13^C-NMR spectrum confirmed the alkylation reaction through the chemical shifts at δ_C_ 20.94 ppm attributed to the methyl groups and the carbon signals of the methylene groups were recorded at δ_C_ 25.88, 27.61, 33.66 and 63.63 ppm. The signals corresponding to the **C**=N and **C**=O carbons appeared at δ_C_ 164.86 and 170.99 ppm, respectively.

Hydrazinolysis of the bis-ester **2** with hydrazine hydrate, in refluxing ethanol for 6 h, furnished the corresponding bis-acid hydrazide **3** which upon condensation with alkyl/aryl isothiocyanates, in ethanol under reflux for 6–8 h, led to the precursor’s bis-acid thiosemicarbazides **4**–**6** in good yields (86%–89%).

The success of the hydrazinolysis reaction was confirmed by IR, ^1^H-NMR and ^13^C-NMR analysis of compound **3**. In the IR spectrum, the characteristic NH and NH_2_ groups of the hydrazide moiety were observed at 3250–3400 cm^−1^. Its ^1^H-NMR spectrum showed a quintet at δ_H_ 1.89–1.96 ppm characteristic for the methylene group CH_2_C**H**_2_CH_2_. The remaining methylene protons of C**H**_2_CO and SC**H**_2_ groups resonated as two triplets at δ_H_ 2.17 and 3.27 ppm, respectively. In addition, the N**H**_2_ and N**H** group were assigned to two singlets appearing at δ_H_ 4.22 and 9.02 ppm, respectively. In the ^13^C-NMR spectrum, the signals belonging to the three carbons of the methylene groups appeared at δ_C_ 18.65, 24.38 and 29.16 ppm, while the C=O amide group showed a signal at δ_C_ 166.82 ppm which provided definitive proof for the formation of the bis-acid hydrazide **3**. The structure of the bis-acid thiosemicarbazides **4**–**6** was elucidated from their spectral analyses. Their IR spectra showed the presence of thiocarbonyl group (**C**=S) by the appearance of new absorption band at 1280–1310 cm^−1^. In their ^1^H-NMR spectra, the disappearance of the hydrazide (N**H**_2_, N**H**) protons and appearance of characteristic thiosemicarbazide NH′s in the range of δ_H_ 7.88–9.90 ppm confirmed the formation of the bis-acid thiosemicarbazides **4**–**6** (see experimental part). The ^1^H-NMR spectrum of the thiosemicarbazide **6** derived from the ethylisothiocyanate revealed the presence of two triplets related to the N**H** that is bonded to the ethyl group at δ_H_ 7.91 and 8.27 ppm, respectively, in a ratio 4 to 1. Additional C**H**_2_ and C**H**_3_ signals belonging to the ethyl group appeared in the aliphatic region. The ^13^C-NMR spectrum supported the formation of compound **6** via the appearance of new characteristic **C**H_2_ and **C**H_3_ groups related to the ethyl substituent in the aliphatic region at δ_C_ 14.46, 18.52 and 38.37, 39.04 ppm, respectively. Moreover, **C**=S group resonated at δ_C_ 175.60 ppm in the ^13^C-NMR spectrum of **6** confirming its presence in the thione form. The remaining carbons were recorded at their corresponding regions.

Thermal intramolecular cyclodehydration of compounds **4**–**6** has been performed in alkaline medium (2N NaOH) to give the corresponding 2,5-bis[(4-aryl/alkyl-2,4-dihydro-1,2,4-triazol-3-thione-5-yl)propylthio]-1,3,4-thiadiazole **7**–**9** in 84%–87% yields (Scheme 2). In contrast, the action of H_2_SO_4_ on the precursors **4**–**6** at 0 °C furnished new 2-aminoalkyl/aryl-1,3,4-thiadiazoles **10**–**12**. On the other hand, compounds **4**–**6** were oxidatively cyclized to the corresponding alkyl/arylamino-1,3,4-oxadiazoles **13**–**15**, in the presence of I_2_/KI in refluxing (4%) NaOH (Scheme 2).

The IR spectra of triazoles **7**–**9** showed common characteristic absorption peaks at 3200–3325 cm^−1^ assigned to (NH), 1267–1295 cm^−1^ (C=S) and 1629–1690 cm^−1^ (C=N) which confirmed the formation of triazole rings. From the ^1^H-NMR spectrum of the N-phenyl derivative **7**, the disappearance of signals related to the N**H** group of the corresponding phenylthiosemicarbazide **4** and appearance of diagnostic triazole-N**H** singlet at δ_H_ 13.72 ppm was a clear evidence for the formation of triazole **7** in its thione form. Moreover, the phenyl protons resonated in the aromatic region as multiplet at δ_H_ 7.41–7.60 ppm. The peaks belonging to the same phenyl group were observed at δ_C_ 128.76–134.18 ppm in the ^13^C-NMR spectrum. The **C**=S signal appeared at δ_C_ 168.10 ppm confirming the predominance of the thione isomer. These results confirmed that compound **4** underwent ring closure to give the corresponding 1,2,4-triazole **7**. The formation of the thiadiazoles **10**–**12** were deduced on the basis of their spectral data, which revealed the disappearance of the carbonyl (C=O) and thiocarbonyl groups in their IR spectra and the appearance of characteristic absorption bands near 1624–1657 cm^−1^ attributed to the C=N group. In addition, the exhibited chemical shifts obtained from their ^1^H-NMR and ^13^C-NMR spectra were all supported by the proposed structures of **10**–**12**. Their ^1^H-NMR spectra confirmed the disappearance of the signals related to the -CON**H** and -N**H**CSN**H**- of their corresponding thiosemicarbazides **4**–**6** around δ_H_ 7.88–9.90 ppm. In the ^1^H-NMR spectrum of the N-ethyl derivative **12**, the characteristic exocyclic N**H** proton at position 2 of the 1,3,4-thiadiazole ring resonated at δ_H_ 7.89 ppm. The aliphatic region showed two additional signals at δ_H_ 1.15 and 3.24–3.28 ppm attributed to the ethyl protons. The peaks belonging to the same group were observed at δ_C_ 14.12 and 40.04 ppm in the ^13^C-NMR spectrum. The spectrum also indicated the disappearance of the signals related to the **C**=O and **C**=S groups at δ_C_ 171.09 and 175.60, respectively.

Compound **14** was taken as a model compound to discuss the obtained spectroscopic data used to confirm the formation of the oxadiazoles ring. The structure was confirmed by IR, ^1^H-NMR, ^13^C-NMR and mass spectra. In the IR spectrum, a new characteristic peak appeared at 1639 cm^−1^ for C=N group and the disappearance of C=S at 1310 cm^−1^ of its corresponding starting **5**. In addition, the ^1^H-NMR analysis revealed the disappearance of the signals related to the **H**NCSN**H** and N**H**CO protons at δ_H_ 7.88, 9.12 and 9.67 ppm of the corresponding methyl thiosemicarbazide **5** and only one singlet appeared at δ_H_ 9.90 ppm related to the amino group. All the other aliphatic protons appeared on their expected chemical shifts. The ^13^C-NMR spectrum of **14** indicated the appearance of the 1,3,4-oxadiazole carbons (C=N) at 157.85 and 168.78 ppm.

Intramolecular ring closure of the bis-acid hydrazide **3** using carbon disulfide in the presence of KOH, in refluxing ethanol for 8 h, afforded the corresponding 2-mercapto-1,3,4-oxadiazole-2-thione **16** in 82% yield (Scheme 3). Moreover, cyclocondensation of **3** with aromatic acid chlorides in the presence of P_2_O_5_ furnished the corresponding unfunctionalized 2,5-disubstituted-1,3,4-oxadiazoles **17**–**19** in 89%–91% yields (Scheme 3).

The IR spectrum of the oxadiazole **16** clearly showed the presence of two characteristic bands around 3210–3280 and 1267 cm^−1^ assigned to NH and C=S groups, respectively. In addition, the disappearance of C=O of the hydrazide group in the starting material **3** confirmed its involvement in the cyclocondensation reaction. The formation of the oxadiazole ring in the proposed structure of compound **16** was also established on the basis of its ^1^H-NMR spectrum in which the existence of a characteristic singlet at δ_H_ 13.74 ppm assigned to the N**H** proton confirming the thione form of such compound. Moreover, the disappearance of two singlets assigned to CON**H** and N**H**_2_ also confirmed the success of the oxidative cyclization of the bis-acid hydrazide **3**. The three C**H**_2_ protons were also recorded in their respected area. In addition, the ^13^C-NMR analysis clearly confirmed the formation of the oxadiazole ring in its thione form through the appearance of **C**=S peak at δ_c_ 167.65 ppm. On the other hand, the structures of the oxadiazoles **17**–**19** were elucidated based on their IR spectra that showed the disappearance of the NH, NH_2_, and C=O groups of the starting material **3**, and the appearance of characteristic absorption bands near 3069–3090 cm^−1^ assigned to aromatic protons. The ^1^H-NMR spectra displayed the presence of the aromatic protons in the region of 7.30–7.62 ppm confirming the structure of the oxadiazoles **17**–**19**.

The treatment of the bis-acid hydrazide **3** with carbon disulphide in ethanolic potassium hydroxide solution led to the formation of potassium dithiocarbazinate salt. Its treatment with hydrazine hydrate under reflux for 8 h furnished the desired 2,5-bis[(4-amino-2,4-dihydro-1,2,4-triazol-3-thione-5-yl)propylthio]-1,3,4-thiadiazole (**20**) at 83% yield (Scheme 4). The significant peaks corresponding to the major functional groups were obvious in the IR spectrum of the aminotriazole **20**. The band at 1310 cm^−1^ clearly indicated the presence of the thione group C=S, while the NH and NH_2_ groups appeared as a sharp band at 3220–3360 cm^−1^. In the ^1^H-NMR spectrum of compound **20** carried out in DMSO-*d*_6_, the presence of two diagnostic singlets at δ_H_ 5.32 and 13.34 ppm of the N**H**_2_ and N**H** protons respectively, depicting the presence of the triazole ring in its thione form. The structure of compound **20** was also supported by ^13^C-NMR. The signal at δ_C_ 163.38 ppm is assigned to the carbon of (**C**=N) linkage of triazole ring, while the signal that appeared at 177.64 ppm is assigned to the (**C**=S) group, which is another evidence of the existence of compound **20** in the thione form.

New Schiff bases **21**–**26** were successfully prepared in good yields (90%–94%), through the condensation of the 5-amino-1,2,4-triazole-3-thione **20** and/or bis-acid hydrazide **3** with several benzaldehyde derivatives in refluxing ethanol, in the presence of catalytic amount of hydrochloric acid (Scheme 4).

In the IR spectra of Schiff bases **21**–**26**, the disappearance of the NH_2_ absorption bands and the appearance of sharp absorption bands at 3218–3367 cm^−1^ related to the NH group confirmed the success of the condensation reaction. The spectra also showed the presence of sharp absorption bands around 3050–3094 cm^−1^ characteristic of Sp^2^ hydrogen of the aromatic ring which were absent in the starting material. In the ^1^H-NMR spectra of compounds **21** and **22**, the disappearance of NH_2_ signal and appearance of two singlets at δ_H_ 9.18–9.24 and 13.47–13.52 ppm attributed to H-C=N and NH protons, respectively, supported the proposed structures. In addition, the presence of C=S carbon at δ_C_ 173.41–173.81 ppm confirmed the formation of Schiff bases **21** and **22** in their thione form.

As expected, the ^1^H-NMR and ^13^C-NMR spectra of the synthesized hydrazones **23**–**26** analyzed in DMSO-*d*_6_ confirmed the existence of a mixture of diastereomers, *i.e.*, *E*/cis and *E*/trans for each imino-amide moiety. These results were in agreement with that previously reported for similar hydrazones which were proved to exhibit *E*/*Z* geometrical isomers about the imine bond (**H**C=N) and cis/trans conformers to the carbonyl amide group [26,27]. This could presumably due to the restricted rotation around the C=N bond which could generates different steric rearrangements of hydrazone functionality in the geometric syn and anti-isomers.

Furthermore, the *E*-geometrical isomer and the cis/trans conformers were the more predominant forms in highly polar solvent such as DMSO-*d*_6_, while the *Z*-isomer appeared only in less polar solvents [28,29]. The ^1^H-NMR spectrum of the unsubstituted phenyl derivative **23** showed two different characteristic singlet peaks at δ_H_ 7.97 and 8.15 ppm with a ratio (2:1) and integrated totally for one proton, which is related to the imine proton (HC=N). The spectrum also showed two broad singlets at δ_H_ 11.31and 11.43 ppm integrated for the N**H** group with the same ratio. In addition, the absence of such pairing of signals in the ^1^H-NMR spectrum of the bis-hydrazide **3** compared to the bis-hydrazones **23**–**26** confirmed the formation these compounds as a mixture of *E*/cis and *E*/trans diastereomers. Regarding to the protons of the propyl chain of compound **23**, the ^1^H-NMR spectrum was not so informative, as these peaks normally appear as multiple peaks. In addition to the evidence about the diastereomers formation for compound **23** by ^1^H-NMR, formation was also confirmed by ^13^C-NMR experiments. In the spectrum; each peak of compound **23** appeared as double peaks due to the presence of diasteriomeric mixture. Focusing on the aliphatic carbons this time, the propyl chain has three different carbon atoms that should be seen in the ^13^C-NMR spectrum. Instead of the three expected signals, a total of six signals were shown in the ^13^C-NMR spectrum reflecting the existence of diastereomers. The ^13^C-NMR spectrum also revealed the presence of two sets of signals in the downfield belonging to **C**=N and **C**=O groups of *E*/cis and *E*/trans diastereomers at δ_C_ 164.98, 167.63 and 167.63, 173.40 ppm, respectively.

### 2.2. Biology

#### 2.2.1. Antimicrobial Activity

All of the newly synthesized compounds were evaluated for their *in vitro* growth inhibitory activities against a panel of standard strains of pathogenic microorganisms including three Gram-positive bacteria, three Gram-negative bacteria, and three strains of fungi. The antimicrobial studies were assessed by minimum inhibitory concentration (MIC) using the broth dilution method [30,31]. MIC is the highest dilution of a compound which shows clear fluid with no development of turbidity.

Antibacterial and antifungal screening revealed that some of the tested compounds exhibited good to excellent activities at concentrations ranging between 4–62.5 μg/mL.

Diethyl 4,4′-[(1,3,4-thiadiazol-2,5-diyl)bis(sulfanediyl)]dibutanoate (**2**) and its hydrazide derivative **3** displayed good activity against Gram-positive and Gram-negative bacterial strains at MIC 16–31.25 μg/mL and moderate activity towards fungal strains at MIC 31.25–62.5 μg/mL. Evaluating the antimicrobial activity of the synthesized 2,2′-(2,2′-(3,3′-(1,3,4-thiadiazol-2,5-diyl)bis-(sulfanediyl)bis-(propane-3,1-diyl)bis-(hydrazine-2,1-diyl)bis-(*N*-aryl/alkyl-2-oxoethanethioamide) **4**–**6**, revealed that compounds exhibited good to moderate antibacterial activity at 16–31.25 μg/mL while their antifungal activity was significantly diminished. Nonetheless, 1,2,4-triazole carrying phenyl and/or alkyl substitution at N-4 **7**–**9** showed excellent antibacterial and antifungal activities against all examined bacterial and fungal strains at 8–16 μg/mL (Table 1).

On other hand, the antimicrobial activity of the thiadiazoles **10**–**12** revealed that all the tested compounds showed comparatively good activity against all bacterial and fungal strains at 8–31.25 μg/mL.

Moreover, 2-amino-1,3,4-oxadiazole derivatives **13**–**15** showed good and greater antibacterial activity against Gram-positive at MIC 8–16 μg/mL. Furthermore, compounds **13**–**15** exhibited moderate antifungal activity at MIC 31.25–62.5 μg/mL.

Among the oxadiazoles **16**–**19**, oxadiazole **16** functionalized with thiol group at position 2 exhibited excellent antibacterial activities against all bacterial strains at MIC 4–8 μg/mL and good activity towards fungal strains at MIC 16–31.28 µg/mL (Table 1).

The incorporation of amino and thiol group into 1,2,4-triazole ring as 2,5-bis[(4-amino-2,4-dihydro-1,2,4-triazol-3-thione-5-yl)propylthio]-1,3,4-thiadiazole **20** resulted in enhancing the antimicrobial activities against all examined bacterial and fungal strains at MIC 4–16 μg/mL.

Evaluating the antibacterial activity of the Schiff bases **21**–**26**, divulged compounds more effective against all bacteria strains at MIC 4–16 μg/mL. Particularly, Schiff bases **23**, **24** comprising fluorine atom exhibited excellent inhibition at MIC 4–8 μg/mL against Gram positive bacteria.

**Table 1:** Antimicrobial activity expressed as MIC (μg/mL).

Compound No.	Gram-Positive Organism ^a^			Gram-Negative Organism ^b^			Fungi ^c^		
	*Sp*	*Bs*	*Sa*	*Pa*	*Ec*	*Kp*	*Af*	*Ca*	*GC*
**2**	31.25	31.25	31.35	16	31.25	16	31.25	62.5	31.25
**3**	16	8	31.25	31.25	16	16	31.25	31.25	62.5
**4**	16	31.25	31.25	31.25	31.25	16	62.5	31.25	62.5
**5**	31.25	31.25	16	31.25	16	31.25	125	250	62.5
**6**	31.25	16	16	31.25	31.25	16	125	250	125
**7**	8	8	16	16	16	8	8	8	16
**8**	16	31.25	16	31.25	31.25	16	16	16	31.25
**9**	16	16	8	31.25	16	31.25	16	31.25	16
**10**	8	16	16	31.25	16	16	16	16	8
**11**	16	31.25	31.25	31.25	31.25	16	31.25	16	16
**12**	16	16	31.25	16	16	31.25	16	16	8
**13**	8	8	16	62.5	62.5	31.25	31.25	31.25	62.5
**14**	16	16	16	31.25	125	62.5	31.25	62.5	62.5
**15**	8	16	8	31.25	62.5	31.25	62.5	31.25	31.25
**16**	4	8	4	4	8	8	31.25	16	16
**17**	16	8	16	16	8	16	31.25	16	16
**18**	16	16	16	16	16	8	31.25	16	16
**19**	16	16	8	16	16	16	31.25	16	16
**20**	4	4	8	8	4	4	16	16	8
**21**	16	8	16	16	8	16	31.25	16	16
**22**	8	8	8	16	8	16	31.25	16	16
**23**	8	8	4	8	4	8	31.25	16	16
**24**	8	4	4	8	4	8	31.25	31.25	16
**25**	8	8	16	8	16	8	16	16	31.25
**26**	8	16	8	8	8	16	31.25	16	16
Ciprofloxacin	≤5	≤1	≤5	≤5	≤1	≤1	-	-	-
Fluconazole	-	-	-	-	-	-	≤1	≤1	≤1

^a^ Gram-positive bacteria: *Streptococcus pneumonia*, *Bacillus subtilis*, *Staphylococcus aureus*; ^b^ Gram-negative bacteria: *Pseudomonas aeuroginosa*, *Escherichia coli*, *Klebsiella pneumonia*; ^c^ yeasts: *Aspergillus fumigatus*, *Candida albicans*, *Geotrichum candidum*.

Antifungal screening of Schiff bases **23**–**26** revealed that all compounds showed good antifungal activity against all examined fungal strains at MIC 16–31.5 μg/mL.

Therefore, it was concluded that 1,2,4-triazole moiety exhibits better antibacterial and antifungal activities than 1,3,4-thiadiazole or 1,3,4-oxadiazole moiety. Interestingly, the presence of azomethine linkage in hydrazones was found essential for the high antibacterial and antifungal activities of these compounds.

#### 2.2.2. Antiproliferative Activity

In an effort to expand our investigation into potential biological activities associated with the newly synthesized compounds, an *in vitro* evaluation of their antiproliferative activities was carried out on four different human cancerous cell lines according to the protocol described in the ISO 10993-5 guide [32]. Only compounds shown in Table 2 exhibited a relatively high cytotoxic activity against the examined cancer cell lines and thus can be used as model compounds for the design and synthesis of new anticancer drugs.

Interestingly, changing phenyl and methyl substitution in compounds **10**, **11** and **13**, **14** into ethyl group to yield compounds **12** and **15** appears to significantly potentiate the cytotoxic activities associated with these analogues suggesting a steric factor mediating either transport or molecular interaction of these compounds with cellular targets. The addition of one more Cl atom into the structure of compound **18** to yield compound **19** was successful in almost doubling its activity. More investigation of possible pathways by which these compounds exert their antiproliferative activity should shed light onto potential molecular targets with which the compounds may interact.

**Table 2:** The LD_50_ values (ng/μL) of the examined compounds on four human cancer cell lines*.* Values are expressed as mean ± SD of three experiments.

Compound No.	MCF-7	T47D	Caco II	HeLa
**12**	376± 16	389 ± 12	387 ± 8	391 ± 10
**15**	409± 9	418± 10	418 ± 9	438 ± 9
**18**	673 ± 15	648 ± 11	668 ± 19	690 ± 17
**19**	398 ± 10	397 ± 9	373 ± 10	356 ± 12
**22**	519 ± 11	527 ± 19	502 ± 17	498 ± 23
**25**	863 ± 12	798 ± 18	678 ± 11	562 ± 8

Human breast adenocarcinoma MCF7, Human ductal breast epithelial tumor T47D, Human epithelial colorectal adenocarcinoma Caco II and Human epithelial carcinoma HeLa.

## 3. Experimental Section

### 3.1. General

Commercially available solvents and reagents were purified according to the stander procedures. All melting points were measured on a Melt-Temp apparatus and are uncorrected. Thin layer chromatography (TLC) was performed on aluminum plates Silica Gel 60 F_254_ (E-Merk, layer thickness 0.2 mm) with detection by UV light absorption. The IR spectra were recorded for the compounds in a KBr matrix with a FTIR-8400s-Fourier transform infrared spectrophotometer-Shimadzu. The NMR spectra were measured with Bruker spectrophotometer (400 and 600 MHz) using TMS as internal standard. ESI mass spectral data were obtained by a Finnigan LCQ spectrometer and EI mass spectra were recorded on a Finnigan MAT 95XL spectrometer. Elemental analyses were performed using an elementar Analysen-systeme GmbH-Vario EL III Element Analyzer.

### 3.2. Synthesis and Characterization of 2,5-Dimercapto-1,3,4-thiadiazole *(**1**)*

To a mixture of hydrazine hydrate (20 mmol) in aqueous solution of sodium hydroxide (12 N) (100 mL) was added carbon disulfide (CS_2_) (40 mmol) in ethanol (100 mL) at 0 °C with stirring during 30 min. Then, the mixture was refluxed for 17 h. After cooling, the mixture was acidified with diluted hydrochloric acid (HCl) and the solid formed was washed, dried and recrystallized from ethanol afforded yellow needles, Yield 98%; m.p. 160–162 °C (Lit. m.p. 162–164 °C) [25].

### 3.3. Synthesis and Characterization of Diethyl 4,4′-[(1,3,4-Thiadiazol-2,5diyl)bis(sulfanediyl)]dibutanoate *(**2**)*

A mixture of compound **1** (1 mmol), potassium carbonate (2.2 mmol) in DMF (20 mL) was stirred for 2 h at room temperature, then ethyl bromobutyrate (2.2 mmol) was added and the mixture was heated under reflux for 8 h. The reaction mixture was cooled and poured onto crushed ice. The product was collected by filtration, washed with water, dried and recrystallized from ethanol yielded compound **2** as colorless crystals, Yield 92%; m.p. 95–96 °C. IR (υ, cm^−1^): 1270 (C-O), 1634 (C=N), 1740 (C=O), 2879, 2936 (C-H al). ^1^H-NMR: δ 1.78–1.82 (m, 2H, CH_2_C**H**_2_CH_2_), 1.87–1.93 (m, 2H, C**H**_2_CO), 2.06 (bs, 3H, CH_2_C**H**_3_), 3.32 (t, 2H, *J* = 4 Hz, SC**H**_2_), 4.08–4.12 (q, 2H, *J* = 4 Hz, *J* = 8 Hz, OC**H**_2_) ppm. ^13^C-NMR: δ 20.94 (CH_2_**C**H_3_), 25.88 (CH_2_**C**H_2_CH_2_), 27.61 (**C**H_2_CO), 33.66 (S**C**H_2_), 63.63 (O**C**H_2_), 164.86 (**C**=N), 170.99 (**C**=O) ppm. EI MS (*m*/*z*): 378.49 (M^+^). Anal. Calcd for C_14_H_22_N_2_O_4_S_3_: C 44.42; H 5.86; N 7.40. Found: C 44.28; H 5.99; N 7.23.

### 3.4. Synthesis and Characterization of 4,4′-[(1,3,4-Thiadiazol-2,5-diyl)bis(sulfanediyl)]dibutanehydrazide *(**3**)*

A mixture of ester **2** (10 mmol) in ethanol (50 mL) and hydrazine hydrate (50 mmol) was refluxed for 6 h. After cooling, ethanol was removed under reduced pressure and the product formed was recrystallized from ethanol yielded compound **3** as white solid, Yield 90%; m.p. 189–191 °C. IR (υ, cm^−1^): 1627 (C=N), 1689 (C=O), 3250–3400 (NH, NH_2_). ^1^H-NMR: δ 1.89–1.96 (quin, 2H, CH_2_C**H**_2_CH_2_), 2.17 (t, 2H, *J* = 8 Hz, C**H**_2_CO), 3.27 (t, 2H, *J* = 8 Hz, SC**H**_2_), 4.22 (bs, 2H, N**H**_2_), 9.02 (s, 1H, N**H**) ppm. ^13^C-NMR δ 18.65 (CH_2_**C**H_2_CH_2_), 24.38 (**C**H_2_CO), 29.16 (S**C**H_2_), 147.80 (**C**=N), 166.82 (**C**=O) ppm. EI MS (*m*/*z*): 350.44 (M^+^). Anal. Calcd for C_10_H_18_N_6_O_2_S_3_: C 34.27; H 5.18; N 23.98. Found: C 34.59; H 5.07; N 24.22.

### 3.5. General Procedure for the Synthesis of Thiosemicarbazide Derivatives ***4**–**6***

A mixture of acid hydrazide **3** (10 mmol) in ethanol (50 mL) and the appropriate alkyl/aryl isothiocyanate (22 mmol) was refluxed for 6–8 h. After cooling, the precipitate formed was collected by filtration and recrystallized from ethanol to afford the desired product.

*2,2′-(2,2′-(3,3′-(1,3,4-Thiadiazol-2,5-diyl)bis-(sulfanediyl)bis-(propane-3,1-diyl)bis-(hydrazine-2,1-diyl)bis-(N-phenyl-2-oxoethanethioamide)* (**4**). This compound was obtained as colorless crystals, Yield 89%; m.p. 247–248 °C. IR (υ, cm^−1^): 1292 (C=S), 1648 (C=N), 1675 (C=O), 3209–3350 (NH). ^1^H-NMR: δ 1.96–2.03 (quin, 2H, CH_2_C**H**_2_CH_2_), 2.32 (t, 2H, *J* = 8 Hz, C**H**_2_CO), 3.21 (t, 2H, *J* = 8 Hz, SC**H**_2_), 7.12–7.47 (m, 5H, Ar-**H**), 9.57 (d, 1H, *J* = 4 Hz, N**H**), 9.62 (s, 1H, *J* = 4 Hz, N**H**), 9.90 (s, 1H, N**H**) ppm. ^13^C-NMR: δ 24.64 (CH_2_**C**H_2_CH_2_), 31.95 (**C**H_2_CO), 32.12 (S**C**H_2_), 126.20, 126.45, 128.13, 128.24, 128.30, 128.49, 128.89, 129.12, 130.06, 130.68, 130.95, 134.13, 135.31, 137.31, 142.49 (Ar-**C**, **C**=N), 173.66, 174.90 (**C**=O, **C**=S) ppm. EI MS (*m*/*z*): 620.32 (M^+^). Anal. Calcd for C_24_H_28_N_8_O_2_S_5_: C 46.43; H 4.55; N 18.05. Found: C 46.71; H 4.74; N 17.98.

*2,2′-(2,2′-(3,3′-(1,3,4-Thiadiazol-2,5-diyl)bis-(sulfanediyl)bis-(propane-3,1-diyl)bis-(hydrazine-2,1-diyl)bis-(N-methyl-2-oxoethanethioamide)* (**5**). This compound was obtained as colorless crystals, Yield 86%; m.p. 192–193 °C. IR (υ, cm^−1^): = 1310 (C=S), 1645 (C=N), 1660 (C=O), 3200–3310 (NH). ^1^H-NMR: δ 1.92–1.99 (quin, 2H, CH_2_C**H**_2_CH_2_), 2.27 (t, 2H, *J* = 8 Hz, C**H**_2_CO), 2.84 (d, 3H, *J* = 4 Hz, NC**H**_3_), 3.18 (t, 2H, *J* = 8 Hz, SC**H**_2_), 7.88 (d, 1H, *J* = 4 Hz, N**H**), 9.12 (d, 1H, *J* = 4 Hz, N**H**), 9.67 (bs, 1H, NH) ppm. ^13^C-NMR: δ 24.55 (CH_2_**C**H_2_CH_2_), 30.81 (N**C**H_3_), 31.91 (**C**H_2_CO), 32.01 (S**C**H_2_), 137.30, 142.49 (**C**=N), 171.37, 173.44 (**C**=O, **C**=S) ppm. EI MS (*m*/*z*): 496.45 (M^+^). Anal. Calcd for C_14_H_24_N_8_O_2_S_5_: C 33.85; H 4.87; N 22.56. Found: C 33.89; H 4.75; N 22.38

*2,2′-(2,2′-(3,3′-(1,3,4-Thiadiazol-2,5-diyl)bis-(sulfanediyl)bis-(propane-3,1-diyl)bis-(hydrazine-2,1-diyl)bis-(N-ethyl-2-oxoethanethioamide)* (**6**). This compound was obtained as colorless crystals, Yield 87%; m.p. 259–260 °C. IR (υ, cm^−1^): 1280 (C=S), 1635 (C=N), 1675 (C=O), 3220–3350 (NH). ^1^H-NMR: δ 1.05 (t, 3H, *J* = 8 Hz, CH_2_C**H**_3_), 1.93–2.00 (quin, 2H, CH_2_C**H**_2_CH_2_), 2.30 (t, 2H, *J* = 8 Hz, C**H**_2_CO), 3.30 (t, 2H, *J* = 8 Hz, SC**H**_2_), 3.42–3.48 (m, 2H, NC**H**_2_), 7.91 (t, 0.8H, *J* = 4 Hz, N**H**), 8.27 (bt, 0.2H, N**H**), 9.04 (s, 0.8H, N**H**), 9.10 (s, 0.2H, N**H**), 9.38 (s, 0.1H, N**H**), 9.68 (s, 1H, N**H**) ppm. ^13^C-NMR: δ 14.46, 18.52 (CH_2_**C**H_3_), 24.39, 24.40 (CH_2_**C**H_2_CH_2_), 29.37, 31.83 (**C**H_2_CO), 33.48, 33.55 (S**C**H_2_), 38.37, 39.04 (N**C**H_2_), 164.89, 164.96 (**C**=N), 171.09, 175.60 (**C**=O, C=S) ppm. EI MS (*m*/*z*): 524.30 (M^+^). Anal. Calcd for C_16_H_28_N_8_O_2_S_5_: C 36.62; H 5.38; N 21.35. Found: C 36.88; H 5.48; N 21.08.

### 3.6. General Procedure for the Synthesis of Triazole Derivatives ***7**–**9***

A solution of the appropriate thiosemicarbazide derivative **4**–**6** (1 mmol) in aqueous sodium hydroxide 2 N (10 mL) was refluxed for 6 h. The resulting solution was cooled to room temperature and acidified with diluted hydrochloride acid. The precipitate formed was filtered, washed with water and recrystallized from ethanol to give the desired product.

*2,5-Bis[(4-phenyl-2,4-dihydro-1,2,4-triazol-3-thione-5-yl)propylthio]-1,2,4-triazole* (**7**). This compound was obtained as colorless crystals, Yield 87%; m.p. 290–291 °C. IR (υ, cm^−1^): 1267 (C=S), 1629 (C=N), 3200–3310 (NH). ^1^H-NMR δ 1.74–1.79 (quin, 2H, CH_2_C**H**_2_CH_2_), 2.27 (t, 0.8H, *J* = 6 Hz, C**H**_2_C=N), 2.45–2.54 (m, 3.2H, C**H**_2_C=N, SC**H**_2_), 7.41–7.60 (m, 5H, Ar**-H**), 13.72 (s, 1H, N**H**) ppm. ^13^C-NMR: δ 23.34 (CH_2_**C**H_2_CH_2_), 24.32 (**C**H_2_C=N), 29.72 (S**C**H_2_), 128.76, 129.92, 129.95, 134.18 (Ar-**C**), 152.09, 168.10 (**C**=N, **C**=S) ppm. ESI MS (*m*/*z*): 585.40 (M + H)^+^. Anal. Calcd for C_24_H_24_N_8_S_5_: C 49.29; H 4.14; N 19.16. Found: C 49.02; H 4.25; N 19.37

*2,5-Bis[(4-methyl-2,4-dihydro-1,2,4-triazol-3-thione-5-yl)propylthio]-1,2,4-triazole* (**8**). This compound was obtained as colorless crystals, Yield 84%; m.p. 201–203 °C. IR (υ, cm^−1^): 1289 (C=S), 1640 (C=N), 3225–3265 (NH). ^1^H-NMR: δ 2.07–2.14 (quin, 2H, CH_2_C**H**_2_CH_2_), 2.50 (s, 3H, NC**H**_3_), 2.78 (t, 2H, *J* = 4 Hz, C**H**_2_C=N), 3.21 (t, 2H, *J* = 4 Hz, SCH_2_), 13.52 (s, 1H, N**H**) ppm. ^13^C-NMR: δ 23.60 (CH_2_**C**H_2_CH_2_), 25.16 (N**C**H_3_), 29.62 (**C**H_2_C=N), 31.53 (S**C**H_2_), 152.48, 166.58 (**C**=N, **C**=S) ppm. EI MS (*m*/*z*): 460.88 (M^+^). Anal. Calcd for C_14_H_20_N_8_S_5_: C 36.50; H 4.38; N 24.32. Found: C 36.73; H 4.44; N 24.16.

*2,5-Bis[(4-ethyl-2,4-dihydro-1,2,4-triazol-3-thione-5-yl)propylthio]-1,2,4-triazole* (**9**). This compound was obtained as colorless crystals, Yield 85%; m.p. 213–214 °C. IR (υ, cm^−1^): 1295 (C=S), 1690 (C=N), 3260–3325 (NH). ^1^H-NMR: δ_H_ 1.21(t, 3H, *J* = 6 Hz, CH_2_C**H**_3_), 1.91–1.96 (quin, 2H, CH_2_C**H**_2_CH_2_), 2.46 (t, 1H, *J* = 6 Hz, C**H**_2_C=N), 2.60 (t, 1H, *J* = 6 Hz, C**H**_2_C=N), 2.79 (t, 2H, *J* = 6 Hz, SC**H**_2_), 3.94–3.98 (q, 2H, *J* = 6 Hz, *J* = 12 Hz, NC**H**_2_), 13.49 (s, 1H, N**H**) ppm. ^13^C-NMR: δ 13.84 (CH_2_**C**H_3_), 23.55 (CH_2_**C**H_2_CH_2_), 25.58 (**C**H_2_C=N), 30.13 (S**C**H_2_), 39.56 (N**C**H_2_), 151.96, 166.44 (**C**=N, **C**=S) ppm. ESI MS (*m*/*z*): 489.50 (M + H)^+^. Anal. Calcd for C_16_H_24_N_8_S_5_: C 39.32; H 4.95; N 22.93. Found: C 39.15; H 4.83; N 23.09.

### 3.7. General Procedure for the Synthesis of Thiadiazole Derivatives ***10**–**12***

A mixture of the appropriate thiosemicarbazide derivative **4**–**6** (1 mmol) in cold concentrated sulfuric acid (15 mL) was stirred for 30 min. Then the mixture was allowed to reach room temperature. After stirring for an additional 16 h; the resulting solution was poured into ice-cold water and made alkaline to pH 8 using ammonium hydroxide. The precipitate formed was filtered; washed with water and recrystallized from ethanol to give the desired product.

*2,5-Bis[(2-phenylamino-1,3,4-thiadiazol-5-yl)propylthio]-1,3,4-thiadiazole* (**10**). This compound was obtained as white solid, Yield 82%; m.p. 235–236 °C. IR (υ, cm^−1^): 1624 (C=N), 3224–3292 (NH). ^1^H-NMR: δ_H_ 2.15–2.19 (quin, 2H, CH_2_**CH**_2_CH_2_), 3.02 (t, 2H, *J* = 6 Hz, C**H**_2_C=N), 3.30 (t, 2H, *J* = 6 Hz, SC**H**_2_), 7.49–7.84 (m, 5H, Ar**H**), 10.13 (s, 1H, N**H**) ppm. ^13^C-NMR: δ 28.00 (CH_2_**C**H_2_CH_2_), 28.15 (**C**H_2_C=N), 32.78 (S**C**H_2_), 116.06, 126.44, 140.29, 141.04, 158.09, 163.97, 164.44 (Ar-**C**, **C**=N) ppm. EI MS (*m*/*z*): 584.83 (M^+^). Anal. Calcd for C_24_H_24_N_8_S_5_: C 49.29; H 4.14; N 19.16. Found: C 49.13; H 4.06; N 19.28.

*2,5-Bis[(2-methylamino-1,3,4-thiadiazol-5-yl)propylthio]-1,3,4-thiadiazole* (**11**). This compound was obtained as white solid, Yield 80%; m.p. 222–224 °C. IR (υ, cm^−1^): 1636 (C=N), 3217–3290 (NH). ^1^H-NMR: δ 2.03–2.09 (quin, 2H, CH_2_**CH**_2_CH_2_), 2.89–2.94 (m, 5H, C**H**_2_C=N, NC**H**_3_), 3.21 (t, 2H, *J* = 4 Hz, SC**H**_2_), 9.87 (s, 1H, N**H**) ppm. ^13^C-NMR: δ_C_ 26.89 (CH_2_**C**H_2_CH_2_), 28.34 (**C**H_2_C=N), 31.98 (S**C**H_2_), 38.69 (N**C**H_3_), 155.67, 163.72, 168.31 (C=N) ppm. ESI MS (*m/z*): 461.49 (M + H)^+^. Anal. Calcd for C_14_H_20_N_8_S_5_: C 36.50; H 4.38; N 24.32. Found: C 36.31; H 4.50; N 24.49.

*2,5-Bis[(2-ethylamino-1,3,4-thiadiazol-5-yl)propylthio]-1,3,4-thiadiazole* (**12**). This compound was obtained as white solid, Yield 81%; m.p. 280–281 °C. IR (υ, cm^−1^): 1657 (C=N), 3229–3309 (NH). ^1^H-NMR δ 1.15 (t, 3H, *J* = 6 Hz, CH_2_C**H**_3_), 2.09–2.14 (quin, 2H, CH_2_**CH**_2_CH_2_), 2.49 (t, 2H, *J* = 6 Hz, C**H**_2_C=N), 2.92 (t, 2H, *J* = 6 Hz, SC**H**_2_), 3.24–3.28 (q, 2H, *J* = 6 Hz, *J* = 12 Hz, NC**H**_2_), 7.89 (d, 1H, *J* = 6 Hz, N**H**) ppm. ^13^C-NMR: δ 14.12 (CH_2_**C**H_3_), 28.19 (CH_2_**C**H_2_CH_2_), 28.21 (**C**H_2_C=N), 32.80 (S**C**H_2_), 40.04 (N**C**H_2_), 155.88, 164.39, 168.66 (**C**=N) ppm. EI MS (*m*/*z*): 488.30 (M^+^). Anal. Calcd for C_16_H_24_N_8_S_5_: C 39.32; H 4.95; N 22.93. Found: C 39.19; H 4.83; N 22.85.

### 3.8. General Procedure for the Synthesis of Oxadiazole Derivatives ***13**–**15***

To a solution of the appropriate thiosemicarbazide derivative **4**–**6** (1 mmol) in ethanol (20 mL) was added (5 N) NaOH solution until the thiosemicarbazide completely dissolved. Then, a solution of I_2_/KI (5%) was added dropwise until the iodine color persists. The reaction mixture was heated under reflux for 3 h and then filtered. After cooling, the precipitate formed was filtered, washed with water and recrystallized from ethanol to afford the desired product.

*2,5-Bis[(2-phenylamino-1,3,4-oxadiazol-5-yl)propylthio]-1,3,4-thiadiazole* (**13**). This compound was obtained as colorless crystals, Yield 81%; m.p. 285–286 °C. IR (υ, cm^−1^): 1627 (C=N), 3223–3310 (NH). ^1^H-NMR: δ 1.92–2.03 (m, 2H, CH_2_C**H**_2_CH_2_), 2.55–2.62 (m, 2H, C**H**_2_C=N), 3.27 (t, 2H, *J* = 6Hz, SC**H**_2_), 7.31–7.57 (m, 5H, Ar-**H**), 8.68 (s, 1H, N**H**) ppm. ^13^C-NMR: δ 28.19 (CH_2_**C**H_2_CH_2_), 28.21 (**C**H_2_C=N), 32.80 (S**C**H_2_), 124.26, 140.33, 142.89, 148.98, 155.88 (Ar-**C**), 164.39, 168.66 (**C**=N) ppm. EI MS (*m/z*): 552.89 (M^+^). Anal. Calcd for C_24_H_24_N_8_O_2_S_3_: C 52.15; H 4.38; N 20.27. Found: C 52.32; H 4.49; N 20.01

*2,5-Bis[(2-methylamino-1,3,4-oxadiazol-5-yl)propylthio]-1,3,4-thiadiazole* (**14**). This compound was obtained as colorless crystals, Yield 84%; m.p. 268–269 °C. IR (υ, cm^−1^): 1639 (C=N), 3225–3121 (NH). ^1^H-NMR δ 2.05–2.12 (quin, 2H, CH_2_**CH**_2_CH_2_), 2.96–2.99 (m, 5H, C**H**_2_C=N, NC**H**_3_), 3.24 (t, 2H, *J* = 4 Hz, SC**H**_2_), 9.90 (s, 1H, N**H**) ppm. ^13^C-NMR: δ 27.79 (CH_2_**C**H_2_CH_2_), 28.17 (**C**H_2_C=N), 32.07 (S**C**H_2_), 38.83 (N**C**H_3_), 157.85, 168.78 (**C**=N) ppm. ESI MS (*m*/*z*): 429.78 (M + H)^+^. Anal. Calcd for C_14_H_20_N_8_O_2_S_3_: C 39.24; H 4.70; N 26.15. Found: C 39.41; H 4.53; N 26.27.

*2,5-Bis[(2-ethylamino-1,3,4-oxadiazol-5-yl)propylthio]-1,3,4-thiadiazole* (**15**). This compound was obtained as colorless crystals, Yield 85%; m.p. 247–248 °C. IR (υ, cm^−1^): 1637 (C=N), 3229–3299 (NH). ^1^H-NMR: δ 1.20 (t, 3H, *J* = 4 Hz, CH_2_**CH**_3_), 2.04–2.11 (quin, 2H, CH_2_**CH**_2_CH_2_), 2.97 (t, 2H, *J* = 4 Hz, C**H**_2_C=N), 3.24 (t, 2H, *J* = 4 Hz, SC**H**_2_), 3.33–3.42 (q, 2H, *J* = 4 Hz, *J* = 8 Hz, NC**H**_2_), 9.96 (s, 1H, N**H**) ppm. ^13^C-NMR: δ 13.34 (CH_2_**C**H_3_), 27.78 (CH_2_**C**H_2_CH_2_), 28.16 (**C**H_2_C=N), 32.17 (S**C**H_2_), 40.83 (N**C**H_2_), 157.74, 167.74 (**C**=N) ppm. EI MS (*m*/*z*): 456.80 (M^+^). Anal. Calcd for C_16_H_24_N_8_O_2_S_3_: C 42.09; H 5.30; N 24.54. Found: C 42.33; H 5.17; N 24.36.

### 3.9. Synthesis and Characterization of 2,5-Bis[(3H-1,3,4-oxadiazole-2-thione-5-yl)propylthio]-1,3,4-thiadiazole *(**16**)*

To a solution of acid hydrazide **3** (1 mmol) in ethanol (20 mL), potassium hydroxide (2.2 mmol) in water (5 mL) and carbon disulfide (10 mmol) were added. The reaction mixture was heated under reflux for 8 h. The mixture was cooled, diluted with cold water (20 mL) and acidified with diluted HCl. The precipitate formed was collected by filtration, washed with water and recrystallized from ethanol afforded the desired compound **16** as colorless needles, Yield 82%; m.p. 238–239 °C. IR (υ, cm^−1^): 1267 (C=S), 1692 (C=N), 3210–3280 (NH). ^1^H-NMR: δ 1.92–1.99 (quin, 2H, CH_2_C**H**_2_CH_2_), 2.53 (t, 2H, *J* = 8 Hz, C**H**_2_C=N), 3.12 (t, 2H, *J* = 8 Hz, SC**H**_2_), 13.74 (s, 1H, N**H**) ppm. ^13^C-NMR: δ_C_ 24.03 (CH_2_**C**H_2_CH_2_), 25.19 (**C**H_2_C=N), 31.34 (S**C**H_2_), 151.54, 167.65 (**C**=N, C=S) ppm. EI MS (*m*/*z*): 433.76 (M^+^). Anal. Calcd for C_12_H_14_N_6_O_2_S_5_: C 33.16; H 3.25; N 19.34. Found: C 32.97; H 3.34; N 19.36.

### 3.10. General Procedure for the Synthesis of Oxadiazole Derivatives ***17**–**18***

To a solution of acid hydrazide **3** (1 mmol) in acetonitrile (10 mL) was added the appropriate benzoyl chloride (1.3 mmol), and phosphorous pentoxide (5 mmol). The reaction mixture was stirred at room temperature for 20–30 min. Then, the solvent was removed under pressure and the mixture was dissolved in dichloromethane and washed with water (3 × 50 mL). The organic layer was then dried over sodium sulfate and evaporated under reduce pressure. The obtained solid was recrystallized from ethanol to afford the desired product.

*2,5-Bis[(2-(2-fluoropenyl)-1,3,4-oxadiazol-5-yl)propylthio]-1,3,4-thiadiazole* (**17**). This compound was obtained as white solid, Yield 89%, m.p. 292–294 °C. IR (υ, cm^−1^): 1635 (C=N), 3069–3072 (Ar-H). ^1^H-NMR: δ 1.90–2.04 (m, 2H, CH_2_C**H**_2_CH_2_), 2.53–2.60 (m, 2H, C**H**_2_C=N), 3.24 (t, 2H, *J* = 6 Hz, SC**H**_2_), 7.30–7.49 (m, 3H, Ar-**H**), 7.52 (dd, 1H, *J* = 6 Hz, *J* = 12 Hz, Ar-H) ppm. ^13^C-NMR: δ 28.09 (CH_2_**C**H_2_CH_2_), 28.24 (**C**H_2_C=N), 32.88 (S**C**H_2_), 124.28, 140.36, 142.99, 149.28, 155.48 (Ar-**C**), 164.29, 168.66 (**C**=N) ppm. EI MS (*m*/*z*): 558.35 (M^+^). Anal. Calcd for C_24_H_20_F_2_N_6_O_2_S_3_: C 51.60; H 3.61; N 15.04. Found: C 51.79; H 3.48; N 15.17.

*2,5-Bis[(2-(2-chlorophenyl)-1,3,4-oxadiazol-5-yl)propylthio]-1,3,4-thiadiazole* (**18**). This compound was as white solid, Yield 91%; m.p. 267–268 °C. IR (υ, cm^−1^): 1648 (C=N), 3073–3089 (Ar-H). ^1^H-NMR: δ 1.94–2.10 (m, 2H, CH_2_C**H**_2_CH_2_), 2.56–2.61 (m, 2H, C**H**_2_C=N), 3.29 (t, 2H, *J* = 6 Hz, SC**H**_2_), 7.41–7.57 (m, 4H, Ar-**H**) ppm. ^13^C-NMR: δ 28.19 (CH_2_**C**H_2_CH_2_), 28.32 (**C**H_2_C=N), 32.67 (S**C**H_2_), 124.37, 140.41, 142.86, 149.98, 156.12 (Ar-**C**), 164.26, 167.96 (**C**=N) ppm. EI MS (*m*/*z*): 590.37 (M^+^). Anal. Calcd for C_24_H_20_Cl_2_N_6_O_2_S_3_: C 48.73; H 3.41; N 14.21. Found: C 48.50; H 3.29; N 14.34.

*2,5-Bis[(2-(2,5-dichlorophenyl)-1,3,4-oxadiazol-5-yl)propylthio]-1,3,4-thiadiazole* (**19**). This compound was obtained as white solid, Yield 90%; m.p. 252–254 °C. IR (υ, cm^−1^): 1664 (C=N), 3075–3090 (Ar-H). ^1^H-NMR: δ 1.96–2.12 (m, 2H, CH_2_C**H**_2_CH_2_), 2.54–2.60 (m, 2H, C**H**_2_C=N), 3.32 (t, 2H, *J* = 6 Hz, SC**H**_2_), 7.48–7.55 (m, 2H, Ar-**H**), 7.62 (s, 1H, Ar-H) ppm. ^13^C-NMR: δ 27.99 (CH_2_**C**H_2_CH_2_), 28.83 (**C**H_2_C=N), 33.17 (S**C**H_2_), 125.47, 141.31, 142.66, 150.77, 157.02 (Ar-**C**), 165.12, 166.99 (**C**=N) ppm. EI MS (*m*/*z*): 658.15 (M^+^). Anal. Calcd for C_24_H_18_Cl_4_N_6_O_2_S_3_: C 43.65; H 2.75; N 12.72. Found: C 43.47; H 2.88; N 12.90.

### 3.11. Synthesis and Characterization of 2,5-Bis[(4-amino-2,4-dihydro-1,2,4-triazol-3-thione-5-yl)propylthio]-1,3,4-thiadiazole *(**20**)*

**Step** **1**: Carbon disulfide (3 mL) was added dropwise to a solution of acid hydrazide **3** (1 mmol) in absolute ethanol (20 mL) containing potassium hydroxide (2.2 mmol) at 0 °C. The reaction was stirred at room temperature for 16 h, and then cooled and diluted with diethyl ether. The precipitate was filtered, washed with diethyl ether and dried. The potassium dithiocarbazinate salt was obtained in nearly quantitative yield and used without further purification as it was moisture sensitive.

**Step** **2**: Hydrazine hydrate (2.5 mmol) was added to a suspension of the potassium salt (1 mmol) in water (15 mL) and the mixture was refluxed with stirring for 8 h. After cooling, it was diluted with water then acidified with aqueous hydrochloric acid. The precipitate was filtered, washed with water and recrystallized from ethanol to give the desired product **16** as colorless crystals, Yield 83%; m.p. 203–205 °C. IR (υ, cm^−1^): 1310 (C=S), 1654 (C=N), 3220–3360 (NH, NH_2_). ^1^H-NMR: δ 2.02–2.13 (m, 2H, CH_2_**CH**_2_CH_2_), 2.73–2.77 (m, 2H, C**H**_2_C=N), 2.91–2.98 (m, 2H, SC**H**_2_), 5.32 (s, 2H, N**H**_2_), 13.34 (s, 1H, N**H**) ppm. ^13^C-NMR: δ 23.57 (CH_2_**C**H_2_CH_2_), 25.10 (**C**H_2_C=N), 32.10 (S**C**H_2_), 163.38, 164.79 (**C**=N), 177.64 (**C**=S) ppm. ESI MS (*m*/*z*): 463.44 (M + H)^+^. Anal. Calcd for C_12_H_18_N_10_S_5_: C 31.15; H 3.92; N 30.27. Found: C 31.38; H 3.78; N 30.16.

### 3.12. General Procedure for the Synthesis of Schiff Bases ***21**–**26***

A mixture of compound **20** and/or **3** (1 mmol) in ethanol (20 mL) and the appropriate aromatic aldehyde (2.2 mmol) with drops of hydrochoric acid was refluxed for 4–6 h. After cooling, the product formed was collected by filtration and recrystallized from ethanol to furnish the desired product.

*2,5-Bis[(4-benzylideneamino)-2,4-dihydro-1,2,4-triazol-3-thione-5-yl)propylthio]-1,3,4-thiadiazole* (**21**). This compound was obtained as white solid, Yield 91%; m.p. 186–188 °C. IR (υ, cm^−1^): 1624 (C=N), 1268 (C=S), 3084 (Ar-H), 3288–3329 (NH). ^1^H-NMR: δ 2.06–2.15 (m, 2H, CH_2_**CH**_2_CH_2_), 2.67–2.73 (m, 2H, C**H**_2_C=N), 2.80–2.88 (m, 2H, SC**H**_2_), 7.40–7.53 (m, 3H, Ar-**H**), 7.69–7.79 (m, 2H, Ar-**H**), 9.18 (s, 1H, **H**-C=N), 13.52 (s, 1H, NH triazole) ppm. ^13^C-NMR: δ 23.36 (CH_2_**C**H_2_CH_2_), 25.60 (**C**H_2_C=N), 32.83 (S**C**H_2_), 126.45, 128.79, 129.40, 134.80 (Ar-**C**), 162.08, 163.77, 165.94 (**C**=N), 173.41 (**C**=S) ppm. ESI MS (*m*/*z*): 638.35 (M + H)^+^. Anal. Calcd for C_26_H_26_N_10_S_5_: C 48.88; H 4.10; N 21.92. Found: C 48.98; H 4.26; N 21.77.

*2,5-Bis[(4-(2-bromobenzylideneamino)-2,4-dihydro-1,2,4-triazol-3-thione-5-yl)-propylthio]-1,3,4-thiadiazole* (**22**). This compound was obtained as white solid, Yield 92% yield; m.p. 206–208 °C. IR (υ, cm^−1^): 1639 (C=N), 1247 (C=S), 3069 (Ar-H), 3214–3344 (NH). ^1^H-NMR: δ 2.02–2.11 (m, 2H, CH_2_**CH**_2_CH_2_), 2.63–2.71 (m, 2H, C**H**_2_C=N), 2.77–2.85 (m, 2H, SC**H**_2_), 7.45–7.58 (m, 2H, Ar-**H**), 7.73–7.82 (m, 2H, Ar-**H**), 9.24 (s, 1H, **H**-C=N), 13.47 (s, 1H, NH triazole) ppm. ^13^C-NMR: δ 23.68 (CH_2_**C**H_2_CH_2_), 25.05 (**C**H_2_C=N), 32.64 (S**C**H_2_), 124.61, 127.80, 128.34, 131.87, 133.13, 141.80 (Ar-**C**), 162.34, 164.18, 165.86 (**C**=N), 173.89 (**C**=S) ppm. ESI MS (*m*/*z*): 793.69 (M + H)^+^. Anal. Calcd for C_26_H_24_Br_2_N_10_S_5_: C 39.20; H 3.04; N 17.58. Found: C 39.41; H 2.86; N 17.45.

*4,4′-(1,3,4-Thiadiazol-2,5-diyl)bis-(sulfanediyl)bis-(N′-(4-benzylidene)butanehyd-razide)* (**23**). This compound was obtained as colorless crystals, Yield 93% yield; m.p. 136–138 °C. IR (υ, cm^−1^): 1669 (C=N), 1714 (C=O), 3064 (Ar-H), 3218–3357 (NH). ^1^H-NMR: δ 2.00–2.05 (quin, 2H, CH_2_C**H**_2_CH_2_), 2.34–2.38 (m, 0.7H, C**H**_2_CO), 2.80 (ddd, 1.3H, *J* = 4 Hz, *J* = 8 Hz, C**H**_2_CO), 3.29–3.36 (m, 2H, SC**H**_2_, overlaped with DMSO), 7.39–7.69 (m, 5H, Ar-**H**), 7.97 (s, 0.65H, **H**-C=N), 8.15 (s, 0.35H, **H**-C=N), 11.31 (s, 0.62H, CON**H**), 11.43 (s, 0.32H, CON**H**) ppm. ^13^C-NMR: δ 24.06, 24.64 (CH_2_**C**H_2_CH_2_), 30.76, 32.64 (**C**H_2_CO), 33.51, 33.69 (S**C**H_2_), 126.61, 126.93, 128.75, 129.64, 129.87, 134.20, 134.28, 142.67, 145.93 (Ar-**C**), 164.86, 164.98, 167.63 (**C**=N), 167.63, 173.40 (**C**=O) ppm. EI MS (*m*/*z*): 526.50 (M^+^). Anal. Calcd for C_24_H_26_N_6_O_2_S_3_: C 54.73; H 4.98; N 15.96. Found: C 54.95; H 5.09; N 15.78.

*4,4′-(1,3,4-Thiadiazol-2,5-diyl)bis-(sulfanediyl)bis-(N′-(4-fluorobenzylidene)butane-hydrazide* (**24**). This compound was obtained as colorless crystals, Yield 91%; m.p. 154–155 °C. IR (υ, cm^−1^): 1672 (C=N), 1704 (C=O), 3080 (Ar-H), 3254–3358 (NH). ^1^H-NMR: δ 1.91–2.00 (m, 1.5H, CH_2_C**H**_2_CH_2_), 2.26–2.30 (m, 0.5H, CH_2_C**H**_2_CH_2_), 2.71 (ddd, 1H, *J* = 4 Hz, *J* = 8 Hz, C**H_2_**CO), 3.19–3.37 (m, 3H, C**H_2_**CO, SC**H_2_**_,_ overlaped with DMSO), 7.21 (ddd, 1.5H, *J* = 8 Hz, 12 Hz, Ar-**H**), 7.30 (dd, 0.5H, *J* = 8 Hz, *J* = 12 Hz, Ar-**H**), 7.59–7.67 (m, 1.5H, Ar-**H**), 7.85–7.88 (m, 1H, Ar-**H**, **H**-C=N), 8.07 (s, 0.25H, **H**-C=N), 8.64 (s, 0.25H, **H**-C=N), 11.23 (s, 0.65H, CON**H**), 11.36 (s, 0.35H, CON**H**) ppm. ^13^C-NMR: δ 24.06, 24.64 (CH_2_**C**H_2_CH_2_), 30.65, 30.75 (**C**H_2_CO), 33.50, 33.66 (S**C**H_2_), 130.38, 130.61, 130.70, 130.81, 130.84, 141.52, 144.82 (Ar-**C**), 160.42, 161.57, 164.97, 165.06 (**C**=N), 167.65, 173.38 (**C**=O) ppm. EI MS (*m*/*z*): 562.83 (M^+^). Anal. Calcd for C_24_H_24_F_2_N_6_O_2_S_3_: C 51.23; H 4.30; N 14.94. Found: C 51.42; H 4.39; N 14.98.

*4,4′-(1,3,4-Thiadiazol-2,5-diyl)bis-(sulfanediyl)bis-(N′-(4-trifluoromethylbenzyli-dene)butanehydrazide* (**25**). This compound was obtained as colorless crystals, Yield 94% yield; m.p. 185–186 °C. IR (υ, cm^−1^): 1648 (C=N), 1704 (C=O), 3094 (Ar-H), 3229–3342 (NH). ^1^H-NMR: δ 1.99–2.09 (m, 2H, CH_2_C**H**_2_CH_2_), 2.38–2.41 (m, 0.7H, C**H**_2_CO), 2.84 (ddd, 1.3H, *J =* 8 Hz, 12 Hz, C**H**_2_CO), 3.29–3.37 (m, 2H, SC**H**_2_, overlaped with DMSO), 7.75–7.80 (m, 2H, Ar-**H**), 7.84–7.91 (m, 2H, Ar-**H**), 8.04 (s, 0.65H, **H**-C=N), 8.23 (s, 0.35H, **H**-C=N), 11.51 (s, 0.65H, CON**H**), 11.65 (s, 0.35 H, CON**H**) ppm. ^13^C-NMR: δ 24.02, 24.54 (CH_2_**C**H_2_CH_2_), 30.64, 30.76 (**C**H_2_CO), 32.62, 33.46 (S**C**H_2_), 120.01, 122.71, 125.42, 125.58, 125.62, 127.16, 127.49, 128.12, 129.05, 129.13, 129.44, 129.64, 138.16, 138.30, 140.97, 144.17 (Ar-**C**), 164.85, 164.96, 165.05 (**C**=N), 167.94, 173.64 (**C**=O) ppm. EI MS (*m*/*z*): 662.37 (M^+^). Anal. Calcd for C_26_H_24_F_6_N_6_O_2_S_3_: C 47.12; H 3.65; N 12.68. Found: C 47.31; H 3.79; N 12.49.

*4,4′-(1,3,4-Thiadiazol-2,5-diyl)bis-(sulfanediyl)bis-(N′-(4-bromobenzylidene)but-anehydrazide* (**26**). This compound was obtained as colorless crystals, Yield 90%; m.p. 201–204 °C. IR (υ, cm^−1^): 1692 (C=N), 1709 (C=O), 3050 (Ar-H), 3249–3367 (NH). ^1^H-NMR: δ 1.97–2.09 (quin, 2H, CH_2_C**H**_2_CH_2_), 2.41 (ddd, 0.7H, *J* = 4 Hz, *J* = 8 Hz, C**H**_2_CO), 2.81 (ddd, 1.2H, *J* = 4 Hz, *J* = 8 Hz, C**H**_2_CO), 3.28–3.36 (m, 2H, SC**H**_2_, overlaped with DMSO), 7.41 (ddd, 1H, *J* = 4 Hz, *J* = 8 Hz, Ar-**H**), 7.51 (t, 0.25H, *J* = 4 Hz, Ar-**H**), 7.57–7.69 (m, 1.75H, Ar-**H**), 7.84–7.91 (m, 1H, Ar-**H**), 7.94 (s, 0.5H, **H**-C=N), 8.12 (s, 0.25H, **H**-C=N), 8.71 (s, 0.25H, Ar-**H**), 11.42 (s, 0.65H, CON**H**), 11.56 (s, 0.3H, CON**H**) ppm. ^13^C-NMR: δ 24.01, 24.58 (CH_2_**C**H_2_CH_2_), 30.61, 30.66 (**C**H_2_CO), 32.61, 32.64 (S**C**H_2_), 122.07, 122.15, 125.75, 125.99, 127.25, 128.76, 129.07, 130.75, 130.90, 131.11, 132.18, 132.36, 134.03, 135.98, 136.68, 136.79, 140.99, 144.12 (Ar-**C**), 160.55, 164.93, 165.03 (**C**=N), 167.87, 173.56 (**C**=O) ppm. EI MS (*m*/*z*): 682.21 (M^+^). Anal. Calcd for C_24_H_24_Br_2_N_6_O_2_S_3_: C 42.11; H 3.53; N 12.28. Found: C 42.34; H 3.40; N 12.45.

### 3.13. Biological Activity

#### 3.13.1. Antimicrobial Susceptibility Testing

##### Cell Lines

All synthesized thiadiazoles derivatives **2**–**26** were screened for their *in vitro* antimicrobial activity against the standard pathogenic strains of the Regional Center for Mycology and Biotechnology (RCMB) namely; *Streptococcus pneumonia* RCMB 010010, *Bacillus subtilis* RCMB 010067, *Staphylococcus aureus* RCMB 010025 (Gram-positive bacteria), *Pseudomonas aeuroginosa* RCMB 010043, *Escherichia coli* RCMB 010052, *Klebsiella pneumonia* RCMB 010058 (Gram-negative bacteria), and the yeast-like pathogenic fungus *Aspergillus fumigates* RCMB 02568, *Geotrichum candidum* RCMB 05097 and *Candida albicans* RCMB 05036.

##### Antimicrobial Evaluation Using the MIC Assay

Antimicrobial activity of compounds **2**–**26** was evaluated by broth microdilution method [30,31]. The MIC determination of the synthesized compounds was carried out in side-by-side comparison with ciprofloxacin against Gram-positive and Gram-negative bacteria. The antifungal activity was assayed against yeasts in comparison with fluconazole. The minimum inhibitory concentrations of the compounds were recorded as the lowest concentration of each chemical compounds in the tubes with no turbidity (*i.e*., no growth) of inoculated bacteria/fungi. Test compounds (10 mg) were dissolved in dimethylsulfoxide (DMSO, 1 mL) then diluted in culture medium (Mueller-Hinton Broth for bacteria and Sabouraud Liquid Medium for fungi), further progressive dilutions to obtain final concentrations of 1, 2, 4, 8, 16, 31.25, 62.5, 125, 250 and 500 mg·mL^−1^. DMSO never exceeded 1% *v*/*v*. The tubes were inoculated with 105 cfu·mL^−1^ (colony forming unit/mL) and incubated at 37 °C for 24 h. The growth control consisting of media and media with DMSO at the same dilutions as used in the experiments was employed.

#### 3.13.2. Antiproliferative Susceptibility Testing

##### Cell Lines

Human breast adenocarcinoma MCF7, Human ductal breast epithelial tumor T47D, Human epithelial colorectal adenocarcinoma Caco II and Human epithelial carcinoma HeLa cell lines were purchased from American Type Culture Collection (ATCC, Rockville, MD, USA). Cells were cultured in Dulbecco’s modified eagle medium (DMEM) (Invitrogen, Carlsbad, CA, USA) containing 10% heat inactivated fetal bovine serum (HI-FBS) (Invitrogen), 2 mmol·L^−1^ of L-glutamine, 50 U·mL^−1^ of penicillin and 50 μg·mL^−1^ of streptomycin. Cell lines were maintained in an atmosphere of 5% CO_2_ and 95 relative humidity at 37 °C. All cells used in this study were between passages 20 and 40.

##### Cytotoxicity Evaluation Using the MTT Assay

The cytotoxic effects associated with the examined compounds were evaluated according to the protocol described in the ISO 10993-5 guide [32]. In brief, cells were seeded at seeding density of 1 × 10^4^ cells per well in 96-well plates and incubated to allow adhesion for 24 h. The tested compounds were dissolved in dimethylsulfoxide (DMSO and subsequently diluted in culture media (test sample). Final concentration of DMSO was maintained constant in all treatment groups within a given experiment and never exceeded 1%. Three triplicates of each concentration for all tested compounds were evaluated in three independent assays for a total of 9 triplicates.

Initially, culture medium in each well was replaced with 200 μL of either test or control solutions. DMEM samples were employed as negative controls, and 50% DMSO (*v*/*v*) in DMEM as a positive control. Both test and control samples were allowed for 48 h incubation at 37 °C in a 5% CO_2_ incubator. At the end of the exposure period, MTT assay was carried out as previously described [32]. Briefly, viable cell count was determined using the 3-(4,5-dimethylthiazol-2yl)-2,5-diphenyl tetrazolium bromide (MTT) colorimetric assay. The yellow tetrazolium dye [MTT, 3-(4,5-dimethylthiazol-2-yl)-5-(3-carboxymethoxyphenyl)-2-(4-sulfophenyl)-2*H*-tetrazolium, inner salt] was reduced by metabolically active cells into an intracellular purple formazan product. The quantity of formazan product, as determined by the absorbance at 490 nm, is directly proportional to the number of living cells in the culture. Cell viability was calculated based on the measured absorbance relative to the absorbance of the cells exposed to the negative controls, which represented 100% cell viability.

## 4. Conclusions

In conclusion, we have synthesized new biologically significant heterocycles and/or Schiff bases tagged 1,3,4-thiadiazole moiety and evaluated their antimicrobial and antiproliferative activities. Antimicrobial assay results indicated that most of the synthesized thiadiazole derivatives exhibited good to excellent antimicrobial activity. From the experimental results, it could be concluded that the clubbing of 1,3,4-thiadiazole with 1,2,4-triazole nucleus and/or Schiff base azomethine linkage in one scaffold resulted in the formation of new potent antimicrobial agents. From this work, compounds **12**, **15**, **18**, **19**, **22** and **25** proved to be the most promising active molecules which are capable of inhibiting the growth of human cancer cell lines *in vitro*.
